# Supplementary material for: Integrated algorithm combining plasma biomarkers and cognitive assessments accurately predicts brain β-amyloid pathology
Source: Commun Med (Lond). 2023 May 10;3:65. doi: 10.1038/s43856-023-00295-9 (PMC10172320; doi:10.1038/s43856-023-00295-9)
Supplement: Supplementary file 1 — Supplementary Material [file 43856_2023_295_MOESM1_ESM.pdf]

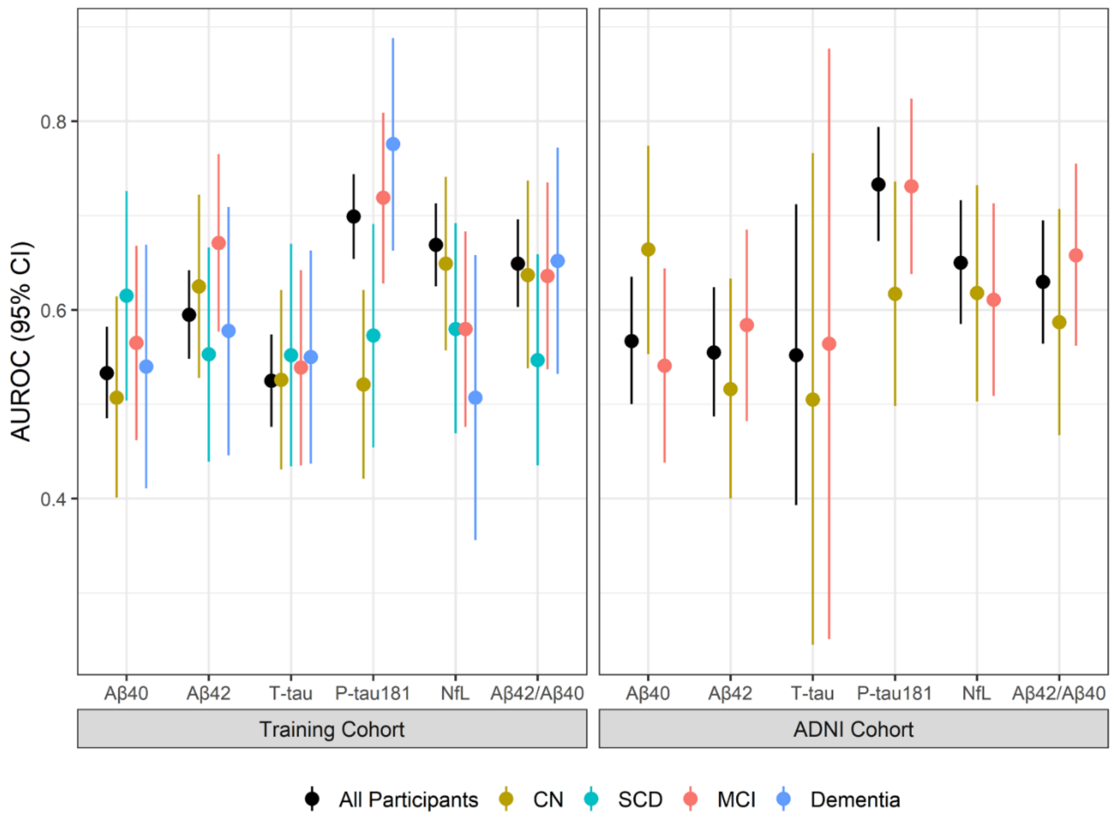

Supplementary Figure 1. Single biomarker performances on Aβ-PET positivity prediction in different diagnostic groups. AUROC values and the corresponding 95% confidence intervals of each individual plasma biomarkers for predicting Aβ-PET positivity in different diagnostic groups (Training Cohort: All participants: n = 609, CN: n = 238, SCD: n = 118, MCI: n = 135, Dementia: n = 118; ADNI Cohort: All participants: n = 284, CN: n = 97, MCI: n = 124) were shown. All values were calculated by receiver operating characteristic (ROC) analyses to evaluate the ability of plasma Aβ40, Aβ42, T-tau, P-tau181, NfL and Aβ42/Aβ40 in identifying Aβ-PET positive independently. Detailed results were shown in **Supplementary Table 3.**

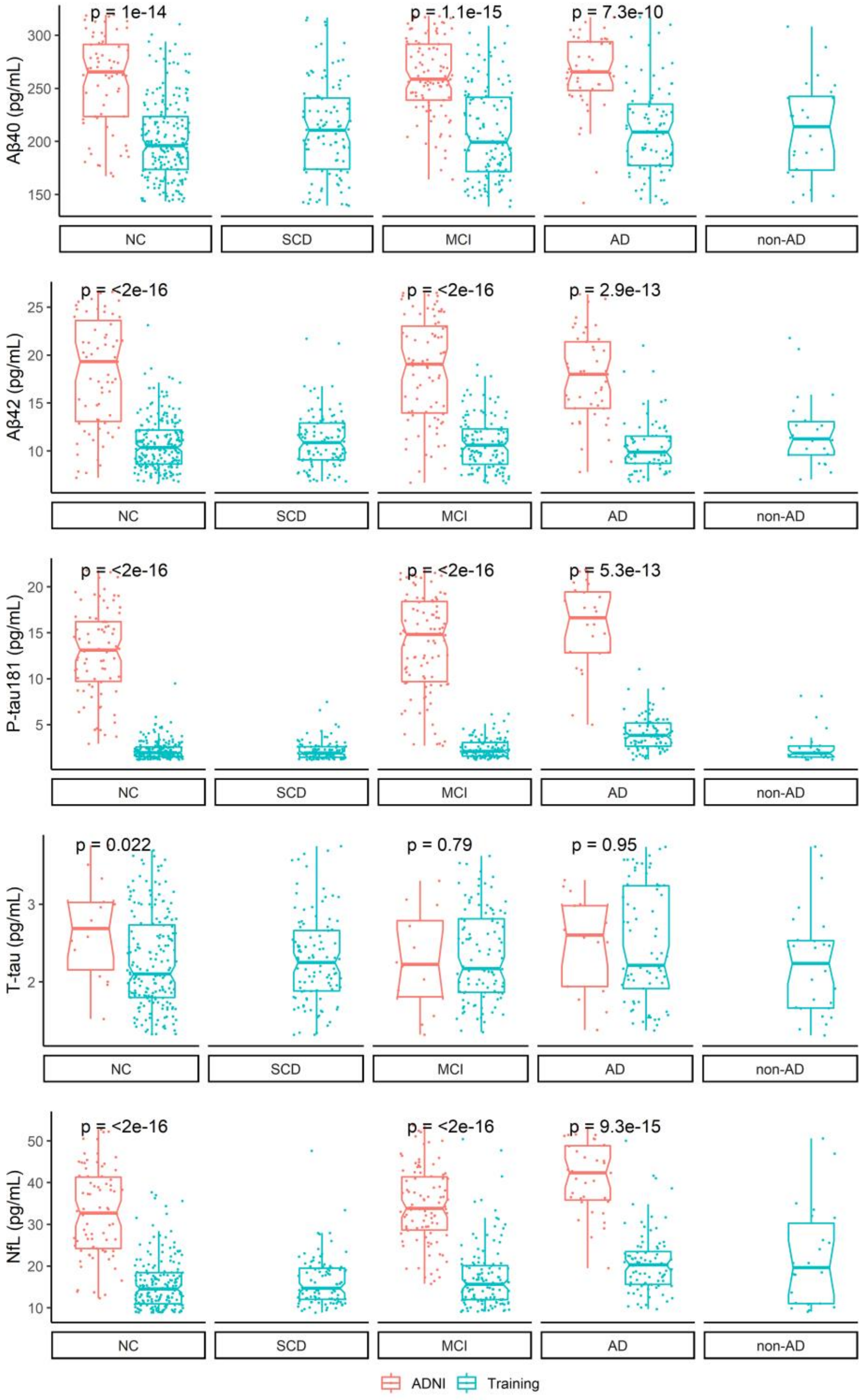

Supplementary Figure 2. Comparison of the levels of Aβ40, Aβ42, P-tau181, T-tau, and NfL in blood between ADNI and training cohort. Y axis shows the raw value of Aβ40, Aβ42, P-tau181, T-tau, and NfL. P value was calculated using Mann-Whitney U test. non-AD: non-Alzheimer's disease dementia. The box shows the 25th percentile, the median, and the 75th percentile of the corresponding data. The whisker lines shows the maximum and minimum value. Minimum value is calculated by Q1 - 1.5*IQR and maximum is calculated by Q3 + 1.5*IQR. IQR: Interquartile range.

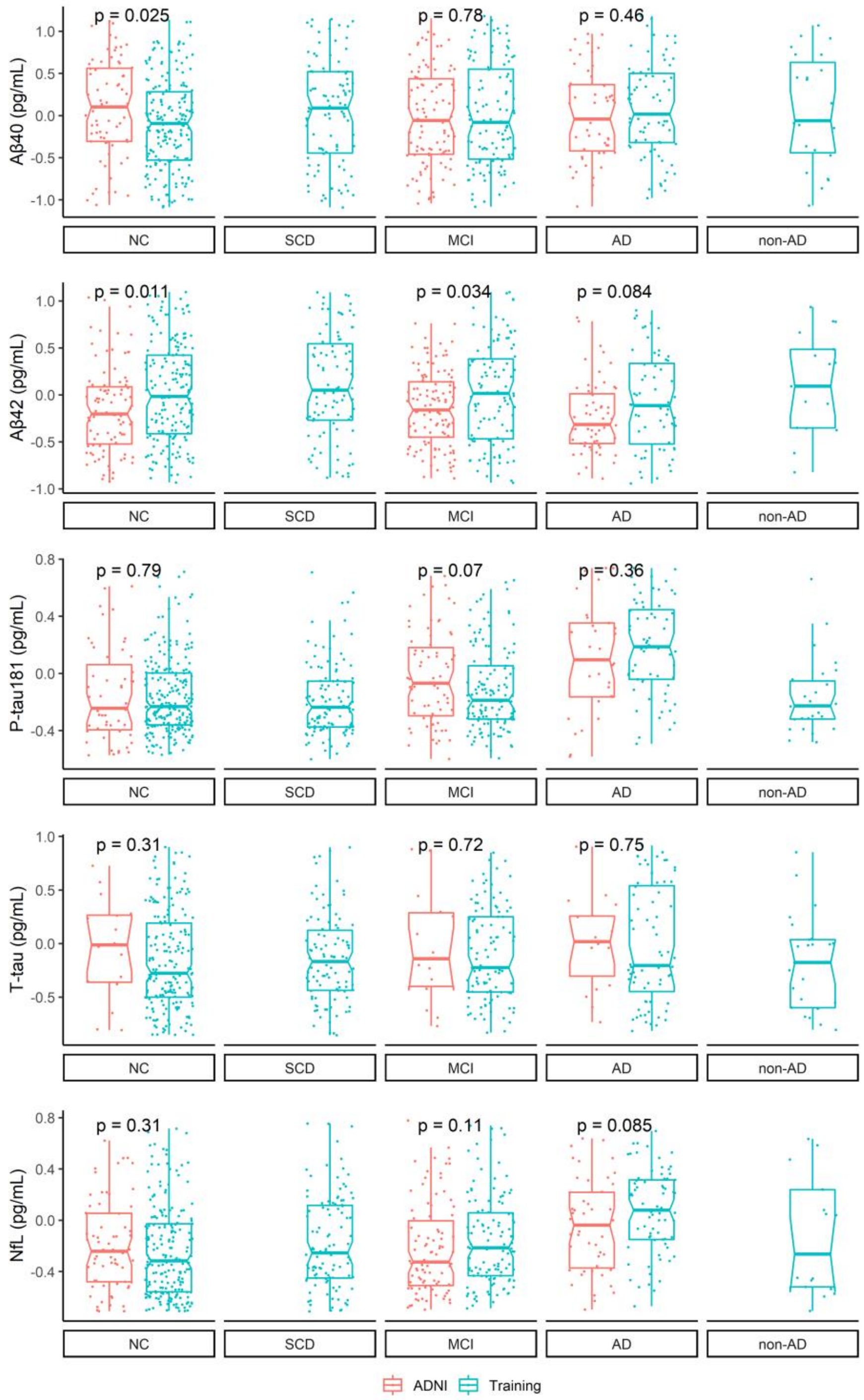

Supplementary Figure 3. Comparison of the levels of scaled Aβ40, Aβ42, P-tau181, T-tau, and NfL in blood between ADNI and training cohort. Y axis shows z-score transformed value within their own dataset. P value was calculated using Mann-Whitney U test. non-AD: non-Alzheimer's disease dementia. The box shows the 25th percentile, the median, and the 75th percentile of the corresponding data. The whisker lines shows the maximum and minimum value. Minimum value is calculated by Q1 - 1.5*IQR and maximum is calculated by Q3 + 1.5*IQR.

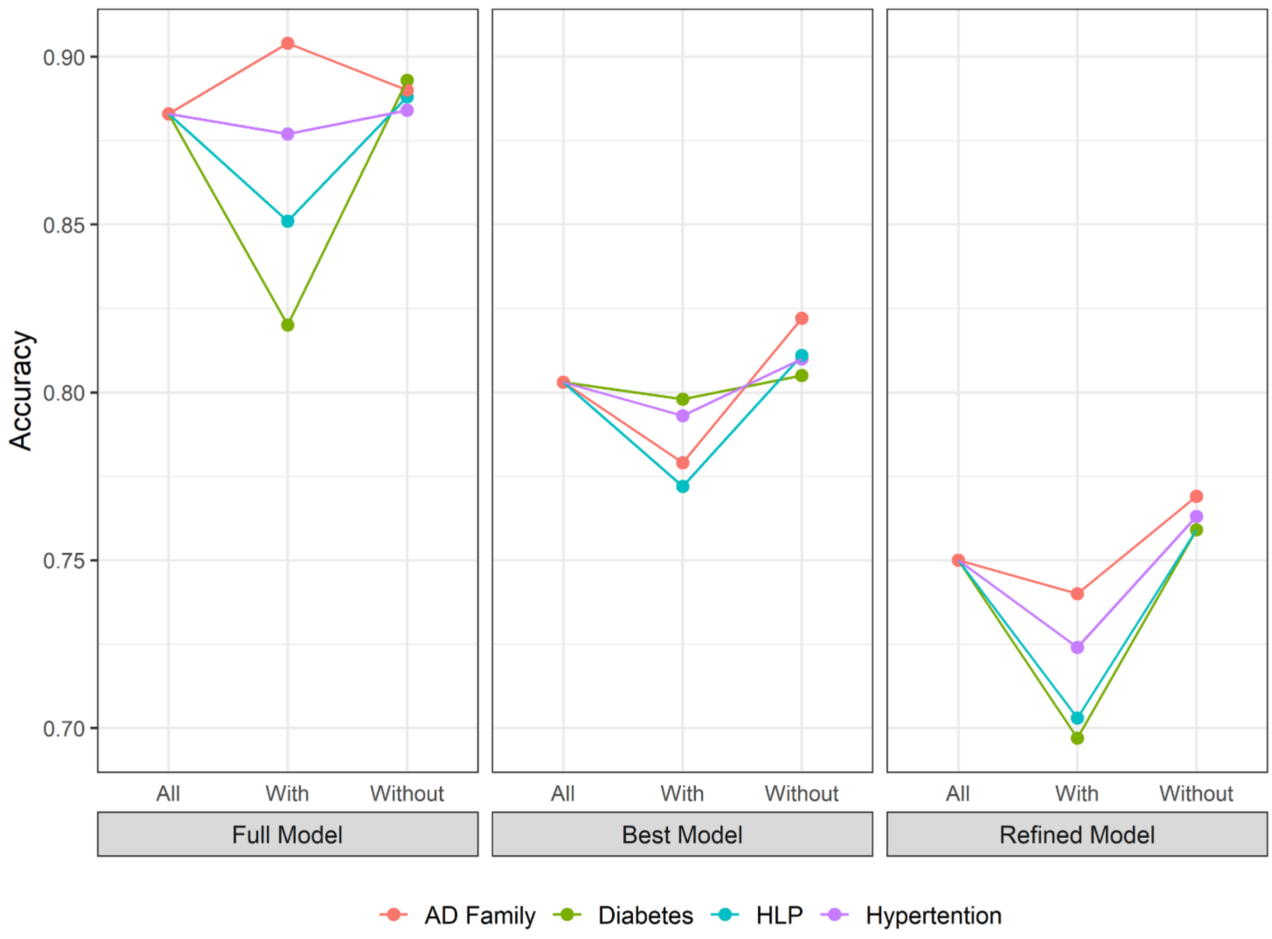

Supplementary Figure 4. Model performances in patients with comorbidities and family histories. Accuracy of the established models for predicting Aβ-PET positivity in participants associated with comorbidities and family history were shown. (AD Family History: n = 382, Diabetes: n = 566, HLP: n = 566, Hypertension: n = 566).

Supplementary Table 1. Plasma biomarker levels in different diagnostic groups by PET status

		All patients		CN		SCD		MCI		Dementia	
		**Aβ-**	**Aβ+**	**Aβ-**	**Aβ+**	**Aβ-**	**Aβ+**	**Aβ-**	**Aβ+**	**Aβ-**	**Aβ+**
**Training Cohort**	**N**	**N = 385 (63.22%)**	**N = 224 (36.78%)**	**N = 192 (80.67%)**	**N = 46 (19.33%)**	**N = 82 (69.49%)**	**N = 36 (30.51%)**	**N = 82 (60.74%)**	**N = 53 (39.26%)**	**N = 29 (24.58%)**	**N = 89 (75.42%)**
	**Aβ40 (pg/mL)[a]**	195.0 (60.2)	198.2 (64.5)	191.8 (57.1)	185.8 (87.4)	197.7 (74.3)	216.0 (57.8)*	200.3 (52.6)	189.9 (58.0)	194.0 (57.1)	202.4 (55.0)
	**Aβ42 (pg/mL)[a]**	10.4 (3.4)	9.2 (3.5)***	10.3 (3.1)	8.5 (4.2)**	10.0 (3.8)	10.5 (3.2)	11.0 (2.9)	9.0 (3.1)***	10.2 (4.8)	9.1 (3.5)
	**T-tau (pg/mL)[a]**	2.4 (1.0)	2.6 (1.8)	2.4 (1.1)	2.6 (2.4)	2.4 (0.9)	2.5 (1.1)	2.4 (1.0)	2.8 (2.3)	2.3 (0.8)	2.6 (1.2)
	**P-tau181 (pg/mL)[a]**	2.0 (1.1)	3.3 (5.0)***	2.0 (1.0)	2.1 (1.5)	1.9 (1.2)	2.1 (1.0)	1.8 (0.7)	2.8 (1.3)***	2.5 (1.9)	4.0 (1.9)***
	**NfL (pg/mL)[a]**	15.9 (14.1)	19.9 (13.4)***	13.9 (8.6)	17.6 (9.8)**	14.2 (6.5)	15.6 (6.1)	17.4 (18.0)	18.0 (8.3)	29.7 (30.5)	24.0 (17.9)
	**Aβ42/Aβ40 ratio[a]**	0.0546 (0.015)	0.0482 (0.017)***	0.0550 (0.014)	0.0486 (0.017)**	0.0527 (0.013)	0.0498 (0.012)	0.0570 (0.019)	0.0506 (0.017)**	0.0511 (0.016)	0.0460 (0.017)*
		**Aβ-**	**Aβ+**	**Aβ-**	**Aβ+**	**Aβ-**	**Aβ+**	**Aβ-**	**Aβ+**	**Aβ-**	**Aβ+**
**ADNI Cohort**	**N**	**N = 122 (42.96%)**	**N = 162 (57.04%)**	**N = 61 (62.89%)**	**N = 36 (37.11%)**	**NA**	**NA**	**N = 56 (45.16%)**	**N = 68 (54.84%)**	**N = 5 (7.94%)**	**N = 58 (92.06%)**
	**Aβ40 (pg/mL)[a]**	275.0 (66.3)	288.0 (63.6)	271.9 (74.0)	311.6 (66.1)**	NA	NA	276.9 (59.3)	284.3 (55.0)	291.6 (42.8)	277.8 (68.6)
	**Aβ42 (pg/mL)[a]**	26.7 (17.4)	24.7 (21.5)	24.4 (12.7)	30.7 (38.0)	NA	NA	29.5 (21.8)	23.1 (11.3)	22.8 (5.5)	22.8 (15.6)
	**T-tau (pg/mL)[a]**	2.4 (1.6)	2.7 (1.6)	2.4 (1.6)	2.3 (0.6)	NA	NA	2.5 (2.2)	2.7 (1.9)	2.3 (0.5)	2.8 (1.7)
	**P-tau181 (pg/mL)[a]**	15.8 (11.1)	22.5 (11.4)***	15.7 (9.4)	19.1 (13.5)	NA	NA	15.9 (13.1)	20.5 (9.0)***	15.7 (6.6)	27.1 (11.3)*
	**NfL (pg/mL)[a]**	43.0 (28.4)	53.0 (32.9)***	39.5 (25.9)	49.0 (30.5)	NA	NA	43.4 (28.2)	51.1 (38.6)*	81.5 (36.4)	57.8 (26.3)
	**Aβ42/Aβ40 ratio[a]**	0.0937 (0.044)	0.0865 (0.074)***	0.0865 (0.030)	0.0914 (0.076)	NA	NA	0.1030 (0.056)	0.0810 (0.039)**	0.0789 (0.017)	0.0898 (0.101)

* p-value < 0.05; ** p-value < 0.01, *** p-value < 0.001 (Mann-Whitney Test between PET positive/negative groups)

[a]: Median and Standard Deviation (sd)

Data are shown as mean (s.d.) or n (%). Group comparisons were performed using the Mann-Whitney U test. Note that no SCD patients in the ADNI cohort contained all five biomarkers, thus were excluded from this study.

Supplementary Table 2. The Distribution of *APOE* genotypes in diagnostic groups

Index	All participants (N = 609)	CN (N=238)	SCD (N=118)	MCI (N=135)	AD (N=89)	Non-AD (N=29)
**APOE ε2/ε2 or ε2/ε3, N(%)**	72 (11.8%)	32 (13.4%)	13 (11.0%)	17 (12.6%)	6 (6.7%)	4 (13.8%)
**APOE ε3/ε3, N(%)**	376 (61.7%)	155 (65.1%)	88 (74.6%)	78 (57.8%)	36 (40.4%)	19 (65.5%)
**APOE ε2/ε4 or ε3/ε4, N(%)**	142 (23.3%)	48 (20.2%)	17 (14.4%)	36 (26.7%)	35 (39.3%)	6 (20.7%)
**APOE ε4/ε4, N(%)**	19 (3.1%)	3 (1.3%)	0 (0.0%)	4 (3.0%)	12 (13.5%)	0 (0.0%)

Supplementary Table 3. Single biomarker performances on Aβ-PET positivity prediction in different diagnostic status

		Overall	CN	SCD	MCI	Dementia
Training Cohort	**Aβ40**	0.535 (0.487 – 0.583)	0.506 (0.400 – 0.613)	0.631 (0.521 – 0.740)	0.565 (0.462 – 0.668)	0.540 (0.411 – 0.669)
	**Aβ42**	0.594 (0.546 – 0.641)	0.626 (0.529 – 0.723)	0.566 (0.453 – 0.679)	0.671 (0.577 – 0.765)	0.578 (0.446 – 0.709)
	**T-tau**	0.527 (0.477 – 0.576)	0.528 (0.433 – 0.623)	0.568 (0.451 – 0.685)	0.539 (0.435 – 0.642)	0.550 (0.437 – 0.663)
	**P-tau181**	0.701 (0.656 – 0.746)	0.519 (0.418 – 0.619)	0.589 (0.471 – 0.706)	0.719 (0.628 – 0.809)	0.776 (0.663 – 0.888)
	**NfL**	0.670 (0.626 – 0.714)	0.647 (0.555 – 0.740)	0.589 (0.477 – 0.701)	0.580 (0.476 – 0.683)	0.507 (0.356 – 0.658)
	**Aβ42/Aβ40**	0.652 (0.605 – 0.698)	0.637 (0.538 – 0.736)	0.560 (0.448 – 0.671)	0.636 (0.537 – 0.735)	0.652 (0.532 – 0.772)
ADNI Cohort	**Aβ40**	0.567 (0.500 – 0.635)	0.664 (0.553 – 0.774)	NA	0.541 (0.438 – 0.644)	NA
	**Aβ42**	0.555 (0.487 – 0.624)	0.516 (0.400 – 0.633)	NA	0.584 (0.482 – 0.685)	NA
	**T-tau**	0.552 (0.393 – 0.712)	0.505 (0.245 – 0.766)	NA	0.564 (0.251 – 0.877)	NA
	**P-tau181**	0.733 (0.673 – 0.794)	0.617 (0.498 – 0.736)	NA	0.731 (0.638 – 0.824)	NA
	**NfL**	0.650 (0.585 – 0.716)	0.618 (0.503 – 0.732)	NA	0.611 (0.509 – 0.713)	NA
	**Aβ42/Aβ40**	0.630 (0.564 – 0.695)	0.587 (0.467 – 0.707)	NA	0.658 (0.562 – 0.755)	NA

All values were shown in AUROC values and the corresponding 95% confidence intervals.

Supplementary Table 4. Statistics in models for different diagnostic status

		AUC (95% CI)	Sensitivity	Specificity	PPV	NPV	Accuracy	CV Error
All Patients	**Full Model**	0.935 (0.915 - 0.955)	83.0%	91.4%	84.9%	90.3%	88.3%	0.914
	**Best Model**	0.830 (0.794 - 0.865)	65.6%	88.8%	77.4%	81.6%	80.3%	0.805
	**Refined Model**	0.708 (0.671 - 0.745)	47.8%	90.9%	75.4%	74.9%	75.0%	0.752
CN	**Full Model**	0.957 (0.916 - 0.997)	89.1%	96.4%	85.4%	97.4%	95.0%	1.104
	**Best Model**	0.824 (0.752 - 0.896)	56.5%	93.8%	68.4%	90.0%	86.6%	1.016
	**Refined Model**	0.689 (0.608 - 0.769)	65.2%	68.8%	33.3%	89.2%	68.1%	1.085
SCD	**Full Model**	0.933 (0.875 – 0.991)	86.1%	95.1%	88.6%	94.0%	92.5%	1.162
	**Best Model**	0.817 (0.740 - 0.894)	86.1%	67.1%	53.4%	91.7%	72.9%	1.025
	**Refined Model**	0.817 (0.740 - 0.894)	86.1%	67.1%	53.4%	91.7%	72.9%	1.025
MCI	**Full Model**	0.967 (0.939 - 0.995)	94.3%	92.7%	89.3%	96.2%	93.3%	0.626
	**Best Model**	0.933 (0.888 - 0.978)	88.7%	93.9%	90.4%	92.8%	91.9%	0.491
	**Refined Model**	0.887 (0.829 - 0.944)	88.7%	80.5%	74.6%	91.7%	83.7%	0.666
aMCI	**Full Model**	0.972 (0.942 – 1.00)	89.7%	94.4%	92.1%	92.7%	92.5%	0.448
	**Best Model**	0.958 (0.921 - 0.994)	94.9%	88.9%	86.0%	96.0%	91.4%	0.458
	**Refined Model**	0.890 (0.835 - 0.946)	94.9%	70.4%	69.8%	95.0%	80.6%	0.699
Dementia	**Full Model**	0.971 (0.948 - 0.995)	89.9%	93.1%	97.6%	75.0%	90.7%	1.423
	**Best Model**	0.855 (0.769 - 0.941)	91.0%	72.4%	91.0%	72.4%	86.4%	1.108
	**Refined Model**	0.755 (0.656 - 0.853)	91.0%	58.6%	87.1%	68.0%	83.1%	1.072

PPV: positive predictive value; NPV: negative predictive value.

Supplementary Table 5. Statistics in models for different diagnostic status in ADNI

		AUC (95% CI)	Sensitivity	Specificity	PPV	NPV	Accuracy	CV Error
All Patients	**Full Model**	0.955 (0.931 - 0.979)	92.6%	90.2%	92.6%	90.2%	91.5%	0.777
	**Best Model**	0.883 (0.844 - 0.923)	79.6%	84.4%	87.2%	75.7%	81.7%	0.866
	**Refined Model**	0.750 (0.695 - 0.805)	65.4%	79.5%	80.9%	63.4%	71.5%	0.865
CN	**Full Model**	0.912 (0.847 - 0.977)	88.9%	83.6%	76.2%	92.7%	85.6%	0.926
	**Best Model**	0.855 (0.779 - 0.931)	72.2%	88.5%	78.8%	84.4%	82.5%	1.131
	**Refined Model**	0.713 (0.629 - 0.789)	41.7%	88.5%	68.2%	72.0%	71.1%	1.158
MCI	**Full Model**	0.950 (0.913 - 0.986)	92.6%	87.5%	90.0%	90.7%	90.3%	0.761
	**Best Model**	0.932 (0.889 - 0.974)	80.9%	91.1%	91.7%	79.7%	85.5%	0.681
	**Refined Model**	0.869 (0.806 - 0.933)	83.8%	83.9%	86.4%	81.0%	83.9%	0.623

Supplementary Table 6. Statistics in disease stage prediction models

	AUC (95% CI)	Sensitivity	Specificity	PPV	NPV	Accuracy	CV Error
**AD vs. CN**	0.931 (0.898 - 0.964)	84.3%	91.2%	78.1%	93.9%	89.3%	0.806
**AD vs. SCD**	0.914 (0.873 - 0.956)	84.3%	93.2%	90.4%	88.7%	89.4%	0.617
**AD vs. MCI**	0.916 (0.877 - 0.955)	84.3%	85.9%	79.8%	89.2 %	85.3%	0.839
**SCD vs. MCI**	0.749 (0.691 – 0.806)	71.1%	71.2%	73.8%	68.3%	71.1%	1.096
**CN vs. SCD**	0.738 (0.684 – 0.791)	51.7%	85.3%	63.5%	78.1%	74.2%	1.173
**CN vs. MCI**	0.695 (0.653 – 0.737)	92.6%	36.1%	45.1%	89.6%	56.6%	1.120

Supplementary Table 7. Detailed accuracies of prediction models in patients with comorbidities and family histories

	AD Family History			Diabetes History			HLP History			Hypertension History		
	All patients	With	Without	All patients	With	Without	All patients	With	Without	All patients	With	Without
**Full Model**	88.3%	90.4%	89.0%	88.3%	82.0%	89.3%	88.3%	85.1%	88.8%	88.3%	87.7%	88.4%
**Best Model**	80.3%	77.9%	82.2%	80.3%	79.8%	80.5%	80.3%	77.2%	81.1%	80.3%	79.3%	81.0%
**Refined Model**	75.0%	74.0%	76.9%	75.0%	69.7%	75.9%	75.0%	70.3%	75.9%	75.0%	72.4%	76.3%

Accuracy values were calculated under the optimal Youden threshold of each prediction model.

Supplementary Table 8. Important values for the variables of full model of full data.

MMSE	P-tau181	MoCA_B	Aβ40	Aβ42/40	NFL	AB42
54.17	49.14	41.68	26.22	25.30	23.89	23.67
ACEIII	Edu_yrs	Age	T-tau	APOE	BMI	Sex
21.92	19.51	16.86	15.98	15.54	12.54	2.90

Supplementary Table 9. Important values for the variables of best model of full data.

MMSE	P-tau181	Aβ42/40	Edu_yrs	Age
49.19	37.80	17.41	11.16	6.58

Supplementary Table 10. Important values for the variables of refined model of full data.

MMSE	P-tau181	Aβ42/40	Edu_yrs	Age
48.55	21.47	3.28	2.64	1.71

Supplementary Table 11. Important values for the variables of full model of negative control (NC) data.

Aβ42	Aβ40	NfL	T-tau	Aβ42/40	*APOE*	Age
27.61	22.52	11.64	10.44	8.47	8.37	8.23
BMI	Edu_yrs	P-tau181	MOCA_B	MMSE	ACEIII	Sex
3.91	3.19	2.46	0.48	0.47	0.45	0.32

Supplementary Table 12. Important values for the variables of best model of NC data.

NfL	Age	P-tau181	*APOE*
19.97	8.71	8.03	2.58

Supplementary Table 13. Important values for the variables of refined model of NC data.

NfL	P-tau181	Age
5.68	4.74	2.27

Supplementary Table 14. Important values for the variables of full model of subjective cognitive decline (SCD) data.

Edu_yrs	Aβ40	Aβ42	NfL	Aβ42/40	T-tau	APOE
11.73	11.58	11.12	7.52	4.84	4.22	1.50
P-tau181	ACEIII	*BMI*	Age	MOCA_B	MMSE	
1.44	1.34	1.11	1.06	1.06	0.69	

Supplementary Table 15. Important values for the variables of best model of SCD data.

Edu_yrs	Aβ40
8.10	7.83

Supplementary Table 16. Important values for the variables of refined model of SCD data.

Edu_yrs	Aβ40
8.10	7.83

Supplementary Table 17. Important values for the variables of full model of mild cognitive impairment (MCI) data.

P-tau 181	Aβ40	Aβ42/40	Aβ42	T-tau	*APOE*	Age
22.83	16.17	13.14	12.75	9.82	7.89	3.22
NfL	Sex	Edu_yrs	MOCA B	ACEIII	BMI	MMSE
2.83	2.52	1.64	1.28	0.85	0.75	0.33

Supplementary Table 18. Important values for the variables of full model of MCI data.

P-tau 181	Aβ40	Aβ42/40	*APOE*
22.01	15.46	12.55	8.79

Supplementary Table 19. Important values for the variables of refined model of MCI data.

P-tau 181	Aβ40	Aβ42/40	*APOE*
18.15	11.60	6.77	0.26

Supplementary Table 20. Important values for the variables of full model of amnestic MCI (aMCI) data.

P-tau181	Aβ42	Aβ40	*APOE*	T-tau	Age	Aβ42/40
17.10	11.00	7.67	6.40	4.03	3.65	3.54
BMI	NfL	Edu_yrs	MOCA B	Sex	MMSE	
2.54	1.86	1.31	1.31	0.87	0.60	

Supplementary Table 21. Important values for the variables of best model of aMCI data.

P-tau 181	Aβ42	Aβ40	*APOE*
17.10	11.00	8.33	6.32

Supplementary Table 22. Important values for the variables of refined model of aMCI data.

P-tau 181	Aβ42	Aβ40
13.63	6.55	6.37

Supplementary Table 23. Important values for the variables of full model of dementia data.

P-tau 181	NfL	Aβ42	AGE	Aβ40	Aβ42/40	T-tau
16.52	13.24	6.02	4.82	4.70	4.70	3.60
APOE	BMI	ACEIII	MMSE	Sex	Edu_yrs	MOCA B
2.61	2.08	1.96	1.34	1.08	0.38	0.27

Supplementary Table 24. Important values for the variables of best model of dementia data.

P-tau 181	NfL	AGE	*APOE*
14.85	8.66	4.41	1.17

Supplementary Table 25. Important values for the variables of refined model of dementia data.

P-tau 181	NfL	AGE	*APOE*
11.25	2.02	1.92	0.67
