# Supplementary material for: Integrated algorithm combining plasma biomarkers and cognitive assessments accurately predicts brain β-amyloid pathology
Source: Commun Med (Lond). 2023 May 10;3:65. doi: 10.1038/s43856-023-00295-9 (PMC10172320; doi:10.1038/s43856-023-00295-9)
Supplement: Supplementary file 3 — Description of Additional Supplementary Files [file 43856_2023_295_MOESM3_ESM.pdf]

Description of Additional Supplementary File

File Name: Supplementary Data 1

Description: Baseline Characteristics of the Study Population. a: Median and interquartile range (IQR), P values were tested by Kruskal-Wallis test; b. Subject count and percentage, P values were tested by Chi-square test. *: P value among dementia status < 0.05. CN, cognitively normal controls; SCD, subjective cognitive decline; MCI, mild cognitive impairment; AD, Alzheimer's disease; BMI, body mass index; APOE, apolipoprotein E; ACE-III-CV, Chinese version of Addenbrooke's Cognitive Examination III; MMSE: Mini-mental State Examination; MoCA-B: The Montreal Cognitive Assessment-Basic; LT, Auditory Verbal Learning Test; BNT, Boston Naming Test; AFT, Animal Verbal Fluency Test; STT-A and B, Shape Trail Test Part A and B; ADL, Activities of Daily Living; FAQ, Functional Assessment Questionnaire.
