# Supplementary material for: Integrated algorithm combining plasma biomarkers and cognitive assessments accurately predicts brain β-amyloid pathology
Source: Commun Med (Lond). 2023 May 10;3:65. doi: 10.1038/s43856-023-00295-9 (PMC10172320; doi:10.1038/s43856-023-00295-9)
Supplement: Supplementary file 5 — Reporting Summary [file 43856_2023_295_MOESM5_ESM.pdf]

nature portfolio

Corresponding author(s): Fang Xie, Zhuo Fang, and Qihao Guo

Last updated by author(s): Mar 16, 2023

# Reporting Summary

Nature Portfolio wishes to improve the reproducibility of the work that we publish. This form provides structure for consistency and transparency in reporting. For further information on Nature Portfolio policies, see our Editorial Policies and the Editorial Policy Checklist.

## Statistics

For all statistical analyses, confirm that the following items are present in the figure legend, table legend, main text, or Methods section.

| n/a | Confirmed | |
|---|---|---|
| ☐ | ☒ | The exact sample size (*n*) for each experimental group/condition, given as a discrete number and unit of measurement |
| ☐ | ☒ | A statement on whether measurements were taken from distinct samples or whether the same sample was measured repeatedly |
| ☐ | ☒ | The statistical test(s) used AND whether they are one- or two-sided<br>*Only common tests should be described solely by name; describe more complex techniques in the Methods section.* |
| ☐ | ☒ | A description of all covariates tested |
| ☐ | ☒ | A description of any assumptions or corrections, such as tests of normality and adjustment for multiple comparisons |
| ☐ | ☒ | A full description of the statistical parameters including central tendency (e.g. means) or other basic estimates (e.g. regression coefficient) AND variation (e.g. standard deviation) or associated estimates of uncertainty (e.g. confidence intervals) |
| ☐ | ☒ | For null hypothesis testing, the test statistic (e.g. *F*, *t*, *r*) with confidence intervals, effect sizes, degrees of freedom and *P* value noted<br>*Give P values as exact values whenever suitable.* |
| ☒ | ☐ | For Bayesian analysis, information on the choice of priors and Markov chain Monte Carlo settings |
| ☐ | ☒ | For hierarchical and complex designs, identification of the appropriate level for tests and full reporting of outcomes |
| ☐ | ☒ | Estimates of effect sizes (e.g. Cohen's *d*, Pearson's *r*), indicating how they were calculated |

*Our web collection on statistics for biologists contains articles on many of the points above.*

## Software and code

Policy information about availability of computer code

| | |
|---|---|
| Data collection | The data that support the findings of this study are available from the corresponding author upon reasonable request. |
| Data analysis | The code that support the findings of this study are available from the corresponding author upon reasonable request. |

For manuscripts utilizing custom algorithms or software that are central to the research but not yet described in published literature, software must be made available to editors and reviewers. We strongly encourage code deposition in a community repository (e.g. GitHub). See the Nature Portfolio guidelines for submitting code & software for further information.

## Data

Policy information about availability of data

All manuscripts must include a data availability statement. This statement should provide the following information, where applicable:

- Accession codes, unique identifiers, or web links for publicly available datasets
- A description of any restrictions on data availability
- For clinical datasets or third party data, please ensure that the statement adheres to our policy

The data that support the findings of this study are available from the corresponding author upon reasonable request.

## Human research participants

Policy information about studies involving human research participants and Sex and Gender in Research.

| | |
|---|---|
| Reporting on sex and gender | Sex was not considered in the study design, as it has not been reported to influence Alzheimer's disease. The demographic table included the sex, however, there was no significant difference between any of the disease stages. The sex has been obtained from patients' medical records. Written informed consent were obtained from all the participants or their caregivers. |
| Population characteristics | aged 40 to 85 years and educated more than one year and fully understand the neuropsychological tests |
| Recruitment | Participants were consecutively enrolled from Sixth People's Hospital, Shanghai, China. The inclusion criteria were as follows: (1) aged 40 to 85 years; (2) educated more than one year and fully understand the neuropsychological tests; (3) consent to the blood tests, cranial MRI and 18F-florbetapir PET scan. The exclusion criteria were as follows: (1) significant systemic illness or renal and hepatic dysfunction which may interfere with the results of plasma biomarkers; (2) Individuals with a history of significant neurologic disease and psychiatric disorders; (3) other conditions which may be adversely affecting cognitive function. 609 participants had available data of both blood tests and 18F-florbetapir PET scan within 3 months after blood sampling, and thus were included in the present study. Written informed consents were obtained from all the participants or their caregivers. The ethics committee of Shanghai Jiao Tong University Affiliated Sixth People's Hospital approved this study. |
| Ethics oversight | The ethics committee of Shanghai Jiao Tong University Affiliated Sixth People's Hospital approved this study. |

Note that full information on the approval of the study protocol must also be provided in the manuscript.

# Field-specific reporting

Please select the one below that is the best fit for your research. If you are not sure, read the appropriate sections before making your selection.

☒ Life sciences ☐ Behavioural & social sciences ☐ Ecological, evolutionary & environmental sciences

For a reference copy of the document with all sections, see nature.com/documents/nr-reporting-summary-flat.pdf

# Life sciences study design

All studies must disclose on these points even when the disclosure is negative.

| | |
|---|---|
| Sample size | 609 participants had available data of both blood tests and 18F-florbetapir PET scan within 3 months after blood sampling, and thus were included in the present study. |
| Data exclusions | The exclusion criteria were as follows: (1) significant systemic illness or renal and hepatic dysfunction which may interfere with the results of plasma biomarkers; (2) Individuals with a history of significant neurologic disease and psychiatric disorders; (3) other conditions which may be adversely affecting cognitive function. |
| Replication | All codes in the study were checked and reproduced by multiple authors. All attempts have been successful in reproducing the results. |
| Randomization | Our study is a retrospective study and no experiment group was allocated. Therefore, randomization is not relevant to our study. |
| Blinding | Our study is a retrospective study and does not include intervention on the patients. Therefore, blinding is not relevant to our study. |

# Reporting for specific materials, systems and methods

We require information from authors about some types of materials, experimental systems and methods used in many studies. Here, indicate whether each material, system or method listed is relevant to your study. If you are not sure if a list item applies to your research, read the appropriate section before selecting a response.

## Materials & experimental systems

| n/a | Involved in the study |
|---|---|
| ☒ | ☐ Antibodies |
| ☒ | ☐ Eukaryotic cell lines |
| ☒ | ☐ Palaeontology and archaeology |
| ☒ | ☐ Animals and other organisms |
| ☒ | ☐ Clinical data |
| ☒ | ☐ Dual use research of concern |

## Methods

| n/a | Involved in the study |
|---|---|
| ☒ | ☐ ChIP-seq |
| ☒ | ☐ Flow cytometry |
| ☒ | ☐ MRI-based neuroimaging |
